# The detection and phylogenetic characterization of *Cryptosporidium*, *Cystoisospora*, and *Giardia duodenalis* of cats in South Korea

**DOI:** 10.3389/fcimb.2023.1296118

**Published:** 2023-11-08

**Authors:** Chi Sun Yun, Bo-Youn Moon, Kichan Lee, Su Min Kang, Bok-Kyung Ku, Mi-Hye Hwang

**Affiliations:** Animal Disease Diagnostic Division, Animal and Plant Quarantine Agency, Gimcheon-si, Republic of Korea

**Keywords:** Cryptosporidium, Cystoisospora, Giardia duodenalis, gastrointestinal protozoa parasite, cat infection

## Abstract

**Introduction:**

*Cryptosporidium*, *Cystoisospora*, and *Giardia duodenalis* are gastrointestinal protozoa parasites that cause diarrhea in various animals. However, information regarding the detection and phylogenetic characterization of gastrointestinal protozoa parasites in cats is limited throughout South Korea. Therefore, this study aimed to determine the detection and identify subspecies of gastrointestinal protozoa parasites in cats from South Korea.

**Methods:**

A total of 290 fecal samples were collected from stray, companion, and shelter cats in six provinces. *Cryptosporidium*, *Cystoisospora*, and *G. duodenalis* were identified by PCR. All positive samples were subtyped by PCR and sequencing of *gp60*, *ITS-1*, *tpi*, *bg*, and *gdh*.

**Results:**

The overall detection of gastrointestinal protozoan parasitic infection was 17.93%. *G. duodenalis* was the most prevalent, with 7.93%, followed by *Cystoisospora* spp. (7.24%) and *Cryptosporidium* spp. (4.48%). In addition, *C. felis* (n=10), *C. parvum* (n=2), *C. ryanae* (n=1), *Cystoisospora felis* (n=14), *Cystoisospora suis* (n=5), *Cystoisospora ohioensis* (n=1), *Cystoisospora* spp. were identified in subspecies analysis of positive samples. *C. felis* showed a significant association with diarrhea (7.81%) and living condition (6.04%), and Cystoisospora felis in diarreha (9.38%) according to detection. Through phylogenetic analysis of the tpi, bg, and gdh genes from 23 G. duodenalispositive samples, it was confirmed that the samples of present study belonged to assemblage A, B, C, and D.

**Discussion:**

South Korean cats have a high rate of gastrointestinal protozoan parasites infection with cat-specific Cryptosporidium and Cystoisospora, which are associated with living conditions and diarrhea symptoms. Moreover, zoonotic and other animal-specific subtype of protozoan parasites have been detected in cat feces.

## Background

With the growth of the pet industry in South Korea, interest in animal health, including cats, has increased, and gastrointestinal parasitic infections have received significant attention for the general health of cats (Kwak and Seo, 2020; Moon et al, 2022). The three most prevalent gastrointestinal protozoa parasites in cats are *Cryptosporidium*, *Cystoisospora*, and *Giardia duodenalis*. *Cryptosporidium* is a protozoan parasite that infects the small intestine and can cause diarrhea, abdominal pain, and fever in cats (de Oliveira et al, 2021; Karimi et al, 2023). This parasite is excreted in the feces of infected cats and can contaminate food and water sources, potentially transmitting to other animals and humans (Current and Haynes, 1984; Feng et al, 2018). *Cryptosporidium* infections are a concern in immunocompromised individuals, causing severe and life-threatening digestive disease. In *Cryptosporidium*, *Cryptosporidium felis* cause a cat-specific infection, and *Cryptosporidium parvum*, a zoonoses infectious genus, is identified in cats (Zahedi et al, 2016; Feng et al, 2018). *Cystoisospora* is another protozoan parasite that infects the small intestine of cats and can cause diarrhea, vomiting, and dehydration (Lindsay et al, 1997; Dubey, 2018). While *Cystoisospora* infections are generally mild and self-limiting, they can be more severe in young or immunocompromised cats (Itoh et al, 2013; Dubey, 2018; Scorza et al, 2021). *G. duodenalis* is a flagellated protozoan parasite that infects the small intestine and can cause diarrhea, vomiting, and weight loss in cats (Epe et al, 2010; Feng and Xiao, 2011). *G. duodenalis* infections are particularly concerning as the parasite can persist in the environment and be difficult to eliminate (Janeczko and Griffin, 2010; Maciel and Sabogal-Paz, 2016).

The detection of gastrointestinal protozoa parasites infections in cats in South Korea is not well documented; however, previous studies have suggested that these infections were detected in several location of the country. For example, the identification of 0.6% of *Cryptosporidium* and 3.8% of *Giardia* infections have been reported in shelter cats in Jeju (Kwak and Seo, 2020) and 30.7% of *Giardia* infections in Daejeon (Lee et al, 2022). In addition, *Cystoisospora* infection has been confirmed microscopically using fecal samples from cats in Daegu (Lee et al, 2019), Suwon (Youn et al, 2012), and around major rivers (Ahn et al, 2019). However, since these previous studies were regionally limited, it is difficult to grasp the pattern of protozoa parasitic infection according to the species and district in domestic cats.

The current status of cats in South Korea is complex, with a large population of stray and feral cats living in urban and rural areas (Lee et al, 2010; Hwang et al, 2016). In addition, cats in abandoned animal shelters encounter various challenges, including overcrowding, lack of resources, and risk of disease transmission (Oh et al., 2021). These gastrointestinal protozoa parasites are a concern for the health of cats as well as have zoonotic potential, meaning they can be transmitted from cats to humans (Zahedi et al, 2016; Dixon, 2021). Therefore, understanding the detection and transmission of these parasites in cats is important for feline health as well as public health. Studying the detection of gastrointestinal protozoa parasites in cats in different environments, including strays, pets, and shelters, can provide important insights into the health status of cats in South Korea and inform efforts to improve their welfare. Furthermore, understanding the zoonotic potential of these parasites can inform public health efforts to reduce the risk of transmission to humans. Therefore, this study aimed to determine the detection and phylogenetic patterns of gastrointestinal protozoa parasites in South Korean cats.

## Methods

### Sample collection

Between January and December 2022, 290 fecal samples were collected from stray (n=149), companion (n=17), and shelter cats (n=124) in six provinces. The sample collection date, sex, and location were recorded using application documents. Dead companion and stray cat bodies were submitted to the Animal and Plant Quarantine Agency (APQA, South Korea) to determine the cause of death and digestive lesions and diarrhea were confirmed at this time, whereas in the case of shelter cats, fecal samples were secured with the cooperation of animal shelters in each city and province in 2022. All fecal samples were stored at 4°C until further experimentations.

### DNA extraction

DNA extraction was performed according to a fecal sample-based adaptation of the Maxwell® RSC PureFood GMO Kit (REF AS1600; Promega Co., Madison, WI, USA) developed by Promega. Briefly, 200–500 mg of fecal samples were placed into a 1.5 mL microcentrifuge tube, 500 mL of CTAB Buffer was added with 35 uL of Proteinase K, and the tubes were heated at 70°C for 30 min and 95°C for 10 min after vortexing. Maxwell® RSC Cartridge preparation and loading on the Maxwell RSC Extraction System (Promega, USA) were performed as described by Promega. All samples were eluted in 100 uL of elution buffer. Extracted DNA was stored at -20°C until further applications.

### PCR amplification and molecular identification

Molecular identification of *Cryptosporidium*, *Cystoisospora*, and *G. duodenalis* was performed by extracting DNA with specific target genes from fecal samples using a thermocycler (Takara, Shiga, Japan) (**Table 1**). All samples were screened using *18S rRNA* gene for *Cryptosporidium* and *G. duodenalis*, and *ITS-1* gene for *Cystoisospora* as described by previous reports (Power et al, 2011; Pallant et al, 2015; Shafiei et al, 2016). The PCR were performed for the *gp60* gene for *C. felis* (Koseoglu et al, 2022), and *tpi*, *gdh* and *bg* for *G. duodenalis* (Sulaiman et al, 2003; Lalle et al, 2005; Caccio et al, 2008) to identify their subtypes. A negative template control sample (RNase-free water) was included in each PCR to confirm contamination with the PCR reaction mixture. PCR-positive amplification products were sequenced using forward and reverse primers (Macrogen Inc., Seoul, South Korea). The nucleotide sequences obtained in the present study were aligned and analyzed with reference sequences from GenBank using BioEdit version 7.2.5. The *Cryptosporidium* and *Cystoisospora* and the assemblages and sub-assemblages of *G. duodenalis* were initially identified from the GenBank database (http://blast.ncbi.nlm.nih.gov). The obtained sequences were deposited in GenBank under the accession numbers OQ598555-OQ598563 for *gp60* of *C. felis*, OQ473126, OQ473172-OQ473185, OQ534549 and OQ534551-OQ534555 for *ITS-1* of *Cystoisospora*, OQ442978-OQ442994 for *tpi* of *G. duodenalis*, OQ442958-OQ442969 for *b-giardin* of *G. duodenalis*, and OQ442970-OQ442977 for *gdh* of *G. duodenalis*.

**Table 1:** Primer sequences and PCR conditions used for the molecular identification and characterization of *Cryptosporidium*, *Cystoisospora*, and *Giardia duodenalis*.

Target species	Gene	Primer nucleotide sequences (5'-3')	Size (bp)	Cycling conditions	Ref.
*Cryptosporidium*	*18S rRNA*	F: AGTGACAAGAAATAACAATACAGG	295	2m/96°C, 40 cycles (30s/94°C, 30s/60°C, 60s/72°C), 7m/72°C	Power et al, 2011
* *	* *	R: CCTGCTTTAAGCACTCTAATTTTC			
*Cryptosporidium felis*	*gp60*	F1: TTTCCGTTATTGTTGCAGTTGCA	1,200	PCR1 and PCR2 : 4m/95°C, 35 cycles (30s/95°C, 30s/55°C, 90s/72°C), 7m/72°C	Koseoglu et al, 2022
* *	* *	R1: ATCGGAATCCCACCATCGAAC			
* *	* *	F2: GGGCGTTCTGAAGGATGTAA	900		
* *	* *	R2: CGGTGGTCTCCTCAGTCTTC			
*Cystoisospora*	*ITS-1*	F1: CCGTTGCTCCTACCGATTGAGTG		PCR1 and PCR2 : 60s/94°C, 40 cycles (10s/98°C, 15s/62°C, 60s/68°C), 5m/68°C	Shafiei et al, 2016
* *	* *	R1: GCATTTCGCTGCGTCCTTCATCG			
* *	* *	F2: GATCATTCACACGTGGCCCTTG	450		
* *	* *	R2: GACGACGTCCAAATCCACAGAGC			
*Giardia duodenalis*	*18S rRNA*	F1: CATCCGGTCGATCCTGCC		PCR1 : 15m/95°C, 35 cycles (30s/95°C, 30s/65°C, 60s/72°C), 7m/72°C PCR2 : 15m/95°C, 35 cycles (30s/95°C, 30s/55°C, 60s/72°C), 7m/72°C	Pallant et al, 2015
* *	* *	R1: GTCGAACCCTGATTCTCCG			
* *	* *	F2: GACGCTCTCCCCAAGGAC	170		
* *	* *	R2: CTGCGTCACGCTGCTCG			
* *	*tpi*	AL3543: AAATIATGCCTGCTCGTCG	605	PCR1 and PCR2 : 5m/94°C, 35 cycles (45s/94°C, 45s/50°C, 60s/72°C), 10m/72°C	Sulaiman et al, 2003
* *	* *	AL3546: CAAACCTTITCCGCAAACC			
* *	* *	AL3544: CCCTTCATCGGIGGTAACTT	532		
* *	* *	AL3545: GTGGCCACCACICCCGTGCC			
* *	*gdh*	Gdh1: TTCCGTRTYCAGTACAACTC	754	PCR1 and PCR2 : 60s/94°C, 40 cycles (10s/98°C, 15s/62°C, 60s/68°C), 5m/68°C	Caccio et al, 2008
* *	* *	Gdh2: ACCTCGTTCTGRGTGGCGCA			
* *	* *	Gdh3: ATGACYGAGCTYCAGAGGCACGT	530		
* *	* *	Gdh4: GTGGCGCARGGCATGATGCA			
* *	*bg*	G7: AAGCCCGACGACCTCACCCGCAGTGC)	753	PCR1 : 15m/95°C, 35 cycles (30s/95°C, 30s/65°C, 60s/72°C), 7m/72°C PCR2 : 15m/95°C, 35 cycles (30s/95°C, 30s/55°C, 60s/72°C), 7m/72°C	Lalle et al, 2005
* *	* *	G759: GAGGCCGCCCTGGATCTTCGAGACGAC			
* *	* *	GiarF: GAACGAACGAGATCGAGGTCCG	511		
* *	* *	GiarR: CTCGACGAGCTTCGTGTT			

### Phylogenetic analysis

Phylogenetic analysis was performed with MEGA X (www.megasoftware.net) using the maximum likelihood method. Phylogenetic tree stability was assessed using a bootstrap value of 1,000 replicates. For multilocus genotyping of *G. duodenalis*, the DNA sequences of *tpi*, *bg*, and *gdh* loci were concatenated in MEGA X to form the MLG, and the reference sequences were selected according to previous studies (Feng and Xiao, 2011; Wang et al, 2019; Wu et al, 2022).

### Statistical analysis

All results are expressed as the percentage of isolates. The frequency of detection of gastrointestinal protozoa parasite isolates from fecal samples was statistically compared using the chi-square test or Fisher’s exact test with a 95% confidence interval, followed by Holm’s *post-hoc* test. The *P-value* was calculated, and statistical significance was set at *P< 0.05*.

## Results

### Detection of Cryptosporidium, Cystoisospora, and Giardia duodenalis in fecal samples of cats

The detection of each parasite infection according to region, season, sex, fecal state, and living conditions is shown in **Table 2**. The detection of *Cryptosporidium* spp. was 4.48% (13/290; CI 2.24–4.98), with 3.45% of *C. felis* (10/290; CI 1.52–4.03), 0.69% of *C. parvum* (2/290; CI 0.23–0.88) and 0.34% of *C. ryanae* (1/290; CI 0.06–0.49). *C. felis* showed a statistically significant difference between fecal states and living conditions (*P< 0.05*). However, no statistically significant differences were observed in the infection of *Cryptosporidium* by region, season, and gender.

**Table 2:** The detection of *Cryptosporidium*, *Cystoisospora* and *Giardia duodenalis* in the stray, companion and shelter cats of South Korea.

		No. of isolates (%)								
		*Cryptosporidium*			*Cystoisospora*				*Giardia duodenalis*	Total
		*C. felis*	*C. parvum*	*C. ryanae*	*C. felis*	*C. suis*	*C. ohioensis*	others		
Region	Gyeonggi (n=112)	5 (4.46)	–	–	3 (2.68)	1 (0.89)	–	–	11 (9.82)	17 (15.18)
	Gangwon (n=11)	–	–	–	–	–	–	–	2 (18.18)	2 (18.18)
	Chungcheong (n=43)	2 (4.65)	–	–	3 (6.98)	–	–	–	3 (6.98)	8 (18.60)
	Gyeongsang (n=56)	3 (5.36)	–	–	2 (3.57)	1 (1.79)	–	–	4 (7.14)	10 (17.86)
	Jeolla (n=38)	–	2 (5.26)	1 (2.63)	3 (7.89)	–	–	1 (2.63)	1 (2.63)	7 (18.42)
	Jeju (n=30)	–	–	–	3 (10.00)	3 (10.00)	1 (3.33)	–	2 (6.67)	8 (26.67)
Season	Spring (n=94)	4 (4.26)	1 (1.06)	–	1 (1.06)	–	1 (1.06)	–	3 (3.19)	10 (10.64)
	Summer (n=53)	–	–	1 (1.89)	3 (5.66)	4 (7.55)	–	–	4 (7.55)	10 (18.87)
	Autumn (n=52)	2 (3.85)	–	–	5 (9.62)	1 (1.92)	–	–	6 (11.54)	13 (25.00)
	Winter (n=91)	4 (4.40)	1 (1.10)	–	5 (5.50)	–	–	1 (1.10)	10 (10.99)	19 (20.88)
Gender	male (n=124)	7 (5.65)	1 (0.81)	–	4 (3.23)	2 (1.61)	–	–	12 (9.68)	24 (19.35)
	female (n=120)	3 (2.50)	1 (0.83)	1 (0.83)	7 (5.83)	1 (0.83)	1 (0.83)	–	6 (5.00)	19 (15.83)
	unknown (n=46)	–	–	–	3 (6.52)	2 (4.35)	–	1 (2.17)	5 (10.87)	9 (19.57)
Fecal states	Diarrhea (n=64)	5 (7.81)^*^	–	–	6 (9.38)^*^	2 (3.13)	–	–	3 (4.69)	15 (23.44)
	Noramal (n=226)	5 (2.21)	2 (0.88)	1 (0.44)	8 (3.54)	3 (1.33)	1 (0.44)	1 (0.44)	20 (8.85)	37 (16.37)
Living condition	Stray (n=149)	9 (6.04)^*^	1 (0.67)	–	7 (4.70)	2 (1.34)	–	–	13 (8.72)	29 (19.46)
	Companion (n=17)	–	1 (5.88)	–	–	–	–	–	2 (11.76)	3 (17.65)
	Shelter (n=124)	1 (0.81)	–	1 (0.81)	7 (5.65)	3 (2.42)	1 (0.81)	1 (0.81)	8 (6.45)	20 (16.13)
Total	Total (n=290)	10 (3.45)	2 (0.69)	1 (0.34)	14 (4.83)	5 (1.72)	1 (0.34)	1 (0.34)	23 (7.93)	52 (17.93)
		13 (4.48)			21 (7.24)					

^*^significant difference (P < 0.05) in parasite prevalence within comparison criteria.

The detection of *Cystoisospora* spp. was 7.24% (21/290; 95% CI 4.16–7.50), including 4.83% of *Cystoisospora felis* (14/290; 95% CI 2.77–5.00), 1.72% of *Cystoisospora suis* (5/290; 95% CI 0.79–1.98), 0.34% of *Cystoisospora ohioensis* (1/290; 95% CI 0.06–0.49) and 0.34% of *Cystoisospora* spp. (1/290; 95% CI 0.07–0.49). The detection of *Cystoisospora* infection was not statistically related to region, season, gender, or living conditions. *Cystoisospora felis* infection was affected by fecal state (*P< 0.05*), as diarrhea (6/64, 9.39%) was higher than normal (8/226, 3.54%) feces.

The detection of *G. duodenalis* was 7.93% (23/290; 95% CI 4.06–8.72). *G. duodenalis* was detected in all regions, seasons, gender, fecal states, and living conditions, although no statistical differences were observed in the detection of *G. duodenalis* infection.

Collectively, the results revealed gastrointestinal protozoa parasite infection was confirmed in 52 out of 290 (17.93%; 95% CI 10.24–18.65) fecal samples of cats. Among them, multiple protozoa parasites were detected in five fecal samples: three were co-infected with *Cystoisospora felis* and *G. duodenalis*, one with *C. felis* and *G. duodenalis*, and one with *Cystoisospora felis* and *C. parvum* (data not shown).

### *Cryptosporidium felis* genotype in fecal samples of cats

**Figure 1:**
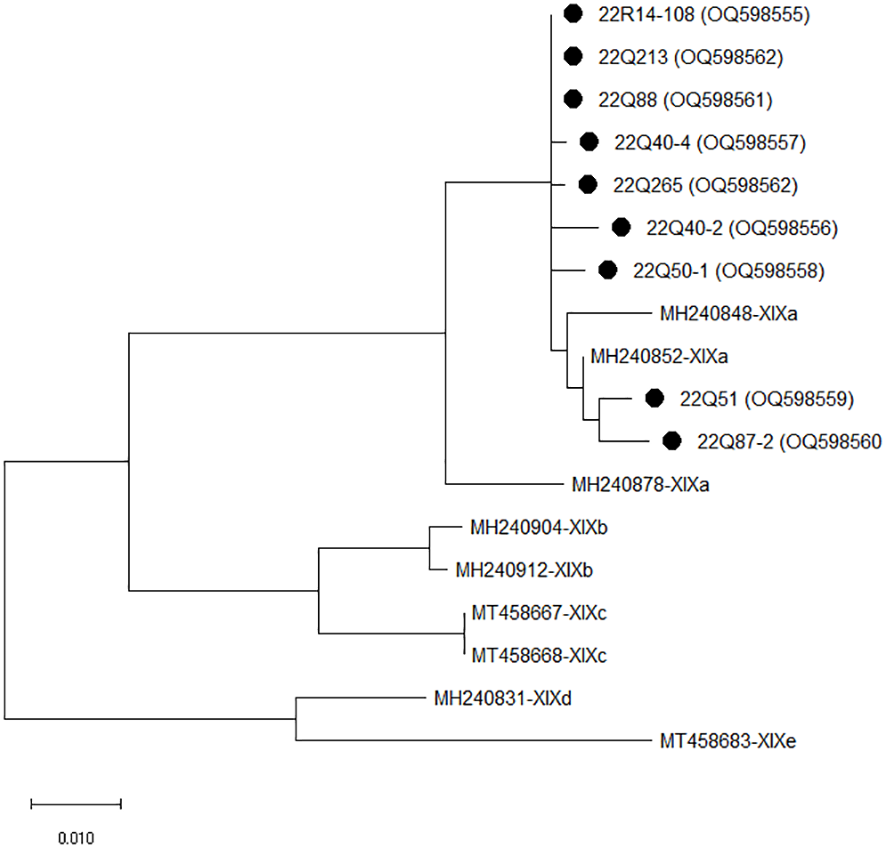
The phylogenetic analysis of *Cryptosporidium felis* based on the *gp60* gene. The phylogenetic tree was constructed using the maximum likelihood method with 1,000 replicates of bootstrap value based on the nucleotide sequence of the *gp60* gene of *C. felis* isolated from stray (Q) and shelter (R) cats. The nucleotide sequences isolated in this study were compared with nucleotide sequences of *C. felis* retrieved from GenBank. Evolutionary analyses were conducted in MEGA X.

The isolates of *C. felis* identified using the *18S rRNA* gene were sequenced using the *gp60* gene for the subtype of *C. felis*. Based on sequence analysis of the *gp60* gene, nine of the 10 isolates were successfully sequenced, while one isolate was non-typable. Four of the sequenced isolates had high homology (98.93 to 99.46%) with the GenBank sequence accession number MH240847. Two isolates had 99.43 to 99.62% homology with MW351825, and the other two isolates had 98.17 to 98.73% homology with MH240865. One isolate showed 97.71% homology to MH240868. Phylogenetic analysis of *C. felis* using *gp60* revealed that all nine isolates in the present study clustered with *C. felis* subtype XIXa (**Figure 1**).

### Phylogenetic analysis of *Cystoisospora* in fecal samples of cats

**Figure 2:**
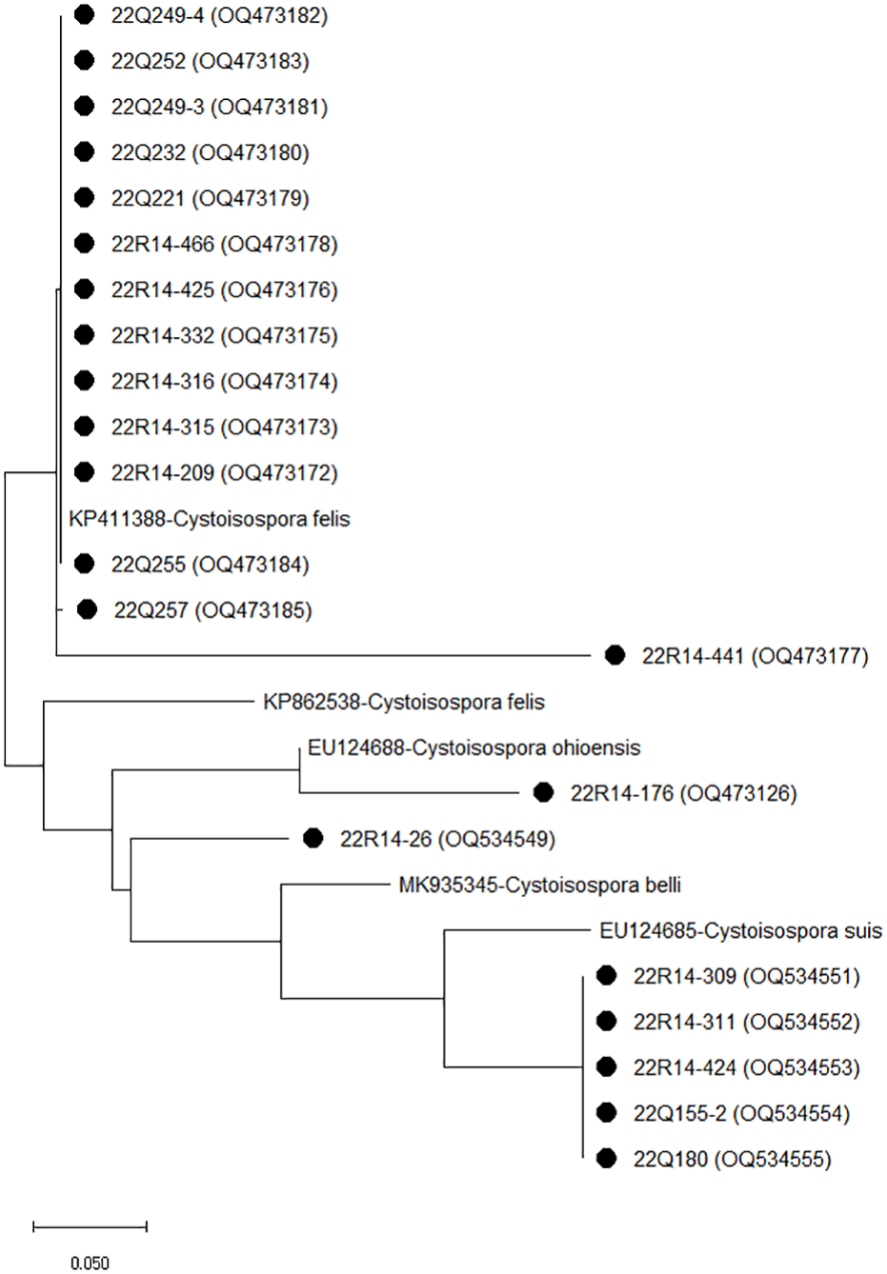
The phylogenetic analysis of *Cystoisospora* based on the *ITS-1* gene. The phylogenetic tree was constructed using the maximum likelihood method with 1,000 replicates of bootstrap value based on the nucleotide sequence of the *ITS-1* gene of *Cystoisospora* isolated from stray (Q) and shelter (R) cats. The nucleotide sequences isolated in this study were compared with nucleotide sequences of *Cystoisospora* retrieved from GenBank. Evolutionary analyses were conducted in MEGA X.

Sequencing and phylogenetic analyses of the amplification products of *ITS-1* in *Cystoisospora* are shown in **Figure 2**. The comparative analysis of 14 *Cystoisospora felis* with the GenBank sequence revealed 99.68 to 100% homology with KP411388. Five *Cystoisospora suis* and one *Cystoisospora ohioensis* isolates showed 90.13% and 90.61% homology with OM870399 and GU292307, respectively. One isolate identified as *Cystoisospora* spp. showed 99.67% homology with MN556343 isolated from tigers and 86.51% with the KP411388 of *Cystoisospora felis*.

### *Giardia duodenalis* assemblages and genotypes in fecal samples of cats

**Figure 3:**
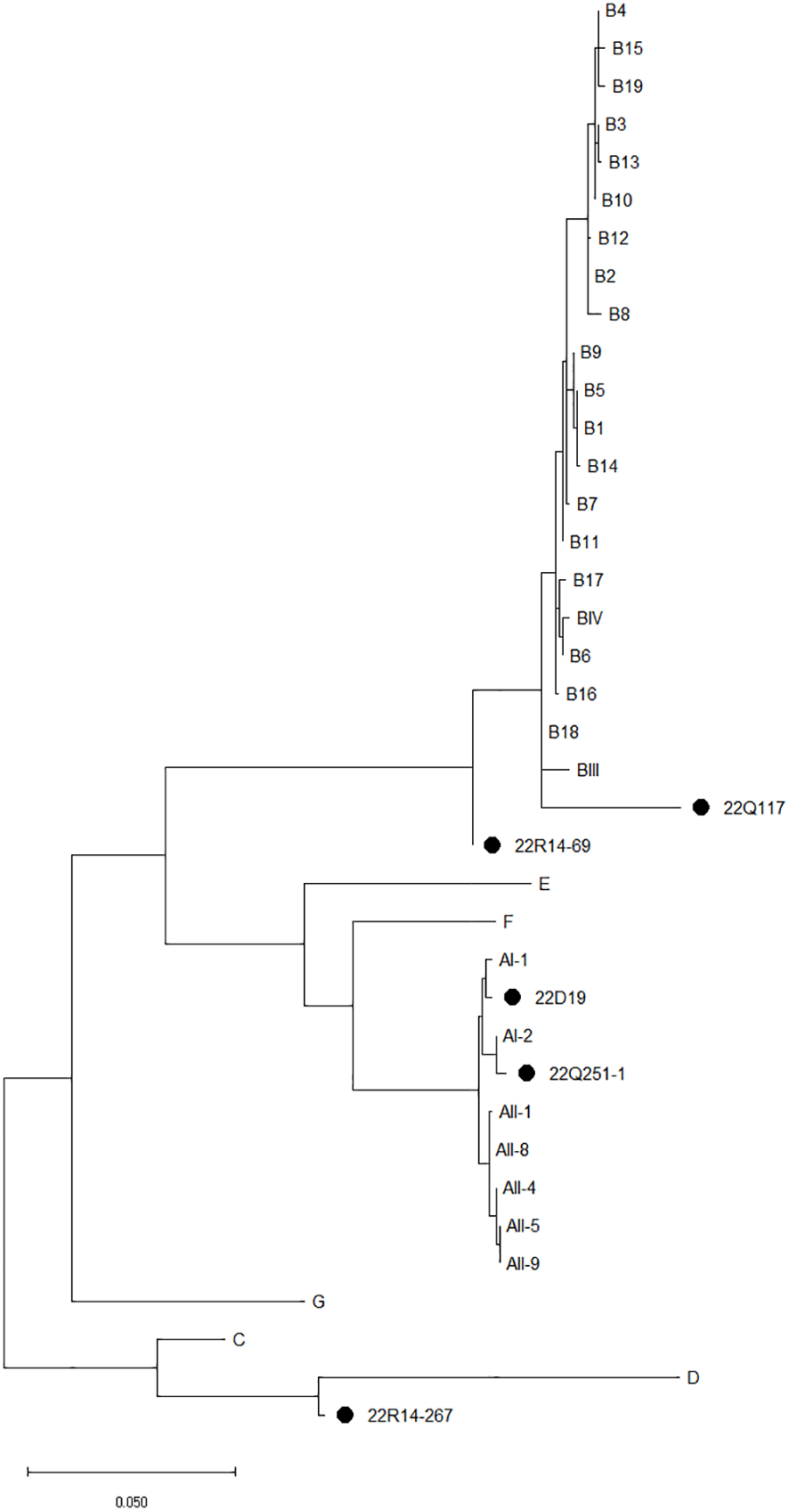
The phylogenetic analysis of *Giardia duodenalis* based on the concatenated multilocus genotypes MLG using *tpi*, *bg*, and *gdh* genes. The phylogenetic tree was constructed using the maximum likelihood method with 1,000 replicates of bootstrap value based on the nucleotide sequence of *tpi*, *bg, and gdh* genes, and MLG of *G. duodenalis* isolated from stray (Q), companion (D), and shelter (R) cats. The nucleotide sequences isolated in this study were compared with those of *G. duodenalis* retrieved from GenBank. Reference sequences for MLG were selected according to previous studies (Feng and Xiao, 2011; Wang et al, 2019; Wu et al, 2022). Evolutionary analyses were conducted in MEGA X.

Sequence analysis of the *tpi*, *bg*, and *gdh* loci revealed an assemblage of *G. duodenalis* (**Table 3**). By sequencing analysis, assemblage A4 (n=4), A5 (n=2), B (n=7), and C (n=4) were identified at the *tpi* locus, and assemblage A (n=2), B5 (n=6), C (n=1), and D (n=3) at the *bg* locus, and assemblage A (n=1), A2 (n=1), B (n=5), and D (n=1) at the *gdh* locus. Among the 23 *G. duodenalis*-positive fecal samples, five were amplified at all *tpi*, *bg*, and *gdh* loci, and concatenated nucleotide sequences were used for MLG (**Figure 3**). The isolates from stray cats were classified as AI-2 and B18. In companions, one isolate of *G. duodenalis* was classified as assemblage AI-1, two isolates from shelter cats were classified as assemblage B, and the other as assemblage D.

**Table 3:** Multilocus genotypes of *tpi, bg*, and *gdh* genes for *Giardia duodenalis* positive isolates.

Living condition	Prevalence	*Giardia duodenalis*			
		tpi	bg	gdh	MLG
Stray	13/149 (8.72 %)	A5	A	A2	AI-2
		B	B5	B	B18
		B	B5		
		A4			
		A4			
		A5			
		B			
		C			
			B5	B	
			C		
			D		
			D		
				B	
Companion	2/17 (11.76 %)	A4	A	A	AI-2
		A4			
Shelter	8/124 (6.45 %)	B	B5	B	B
		C	D	D	D
		B			
		B			
		B	B5		
		C			
		C			
			B5	B	

## Discussion

In the present study, molecular analysis was conducted to identify the detection of infection and the species, and subtypes of *Cryptosporidium*, *Cystoisospora*, and *Giardia duodenalis* of cats in South Korea. Moreover, differences according to the provinces of South Korea, seasons, gender, diarrheal symptoms, and living conditions were analyzed.

With the growing pet industry, the increasing number of cats raised by people, and the large population of stray and abandoned cats in South Korea, concerns about zoonotic parasite infection in cats have been highlighted (Cho et al, 2018). *Cryptosporidium*, *Cystoisospora*, and *Giarida* are the most important gastrointestinal protozoa parasites that infect humans, livestock, and wild animals, which have zoonotic and zooanthroponotic characteristics, and can be transmitted between humans and animals, causing enteric disorders such as diarrhea (Appelbee et al, 2005; Dubey, 2018). Stray cats have an increased risk of transmitting pathogens through contact with humans or infected excrement while roaming the streets (Kwak and Seo, 2020; Mendoza and Otranto, 2023). In shelters, cats raised by humans were included, but kittens and injured cats were also admitted (Cho et al, 2020). At this time, there is a possibility of cross-infection by pathogens in co-breeding environments. These three gastrointestinal protozoa parasites show asymptomatic or weak clinical symptoms in adults; however, they cause acute diarrhea in kitten and young-age cat and can lead to death in severe cases (Tzannes et al., 2008; Epe et al., 2010; Certad et al, 2017). Therefore, considering the risk of infection of humans and young-age cats, it is important to analyze the infection status and characteristics of these protozoa parasites

*Cryptosporidium* has a wide host range, including humans and mammals, and *C. felis* is a cat-specific species that cause diarrhea in cats (Appelbee et al, 2005; Certad et al, 2017). In this study, *C. felis* infection was the most frequent at 3.45% of the total 4.48% *Cryptosporidium* infection. This is consistent with the global *Cryptosporidium* infection rate of 6.0% on average and 3– 9% in Asian countries for cats (Meng et al., 2021; Taghipour et al, 2021). This was higher than the infection rate of *C. felis* recently reported in Jeju, South Korea at 0.6% (Kwak and Seo, 2020), China at 2.3 to 5.02% (Li et al., 2019; Li et al, 2019) and Turkey at 0.0% (Onder et al, 2021). However, the infection rate of *C. felis* is lower than that in Denmark at 6.7% (Rojas-Lopez et al, 2020) and Brazil at 5.4% (de Oliveira et al, 2021). The detection of *Cystoisospora* had an infection rate of 7.24% overall, with a relatively high infection rate of *Cystoisospora felis* at 4.83%. This was higher than the recently reported *Cystoisospora felis* infection rates of 0.53–0.73% in cats in South Korea (Ahn et al, 2019; Lee et al, 2019). Eight cases (8,7%) of *Cystoisospora* infection were previously confirmed by microscopic evaluation in hospitalized and stray cats in South Korea (Youn et al, 2012), but the sampling area was limited to certain areas. *Cystoisospora felis* shows cat-specific infectious properties and has a 12.8% (0.5–76.0%) infection rate in cats worldwide (Lindsay et al, 1997). The present study showed a higher rate of *Cryptosporidium* and *Cystoisospora* infections than previous studies in South Korea, but this varies depending on the region where the survey and sample were obtained. A *gp60* subtyping has been used to analyze the genetic characteristics of *C. felis* (Rojas-Lopez et al, 2020). In the present study, all isolated *C. felis* strains belonged to the XIXa subtype family. The seven *C. felis* in this study showed a high homology rate of 97.71–99.46% with previous studies isolated from humans in the UK (Jiang et al, 2020). The other two isolates from stray cats showed a 99.43–99.62% homology with those isolated from cats in China (Li et al., 2021). In addition, all 14 *Cystoisospora felis* isolates in this study showed 99.68–100% homology with those from cats in the United States (Dubey et al., 2015). As exchanges between countries and civilian travel have become more active, the possibility of protozoa parasitic infection through contact with infected or contaminated humans, animals, water, food, and the environment in other countries has continuously increased (Certad et al, 2017; Kostopoulou et al, 2017). Since the limited information of *C. felis* and *Cystoisospora felis* reported in South Korea, it is unclear whether the *C. felis* and *Cystoisospora felis* in this study were introduced from other countries or originally present in South Korea; thus, continuous observation and further research are required.

In this study, one case each of *C. parvum* infection was detected in stray and companion cats. Cats can be infected with zoonotic *C. parvum*, which can be transmitted to humans and mammals through fecal-oral transmission (Appelbee et al, 2005; Certad et al, 2017; Mendoza and Otranto, 2023). Recently, several cases of *C. parvum* infections in cats have been reported (Li et al., 2019; Tangtrongsup et al, 2020; Taghipour et al, 2021), and the possibility of *C. parvum* infection in cats is expected as well as a carrier that spreads to other animal species in South Korea. The present study showed that one, five, and one cases of *C. ryanae*, *Cystoisospora suis*, and *Cystoisospora ohioensis*, respectively, were detected in cats. *C. ryanae* causes cattle-specific infections (Zahedi et al, 2016). *Cystoisospora suis* and *Cystoisospora ohioensis* are associated with infections in pigs and dog (Lindsay et al., 1997). Moreover, the one *Cystoisospora* spp. isolate showed high homology (99.67%) with MN556343 isolated from tigers in China, which is associated with *Cystoisospora suis* and *Cystoisospora belli* (Chiu et al, 2021). The route of inflow or infection of other animal-specific *Cryptosporidium* and *Cystoisospora* subspecies in cats remains unclear. Information on *Cystoisospora suis* and *Cystoisospora ohioensis* infections in cats is lacking, but one case of cat infection with *C. ryanae* has been reported (Yang et al., 2015). However, some animals, including mice, act as paratenic or reservoir hosts (Dubey, 2018). A previous study showed that *Cystoisospora ohioensis* can remain infectious in mice and be transmitted to other animals (Dubey and Mehlhorn, 1978). Cats are the top predators of small animals, suggesting the possibility of parasite transmission (Mendoza and Otranto, 2023). In a previous study, *Cryptosporidium muris*, which causes rodent-specific infections, was detected in cats that probably ate infected rodents (FitzGerald et al., 2011; Yang et al., 2015). Therefore, although it is uncertain whether the cat is a primary or paratenic host for *C. ryanae*, *Cystoisospora suis*, and *Cystoisospora ohioensis*, it is possible that other animal-specific parasites may be detected in cats by contact with the animal feces, contaminated water near livestock farms, or ingested prey infected with *C. ryanae* and *Cystoisospora* spp as reservoir host. This study showed that rare cases of *C. ryanae*, *Cystoisospora suis* and *Cystoisospora ohioensis* were detected in cats, but further research is needed to determine whether cats act as reservoir host without infection or primary/paratenic host with clinical symptoms.

*G. duodenalis* occurred most frequently among the three parasites, at 7.93%, although there were no significant differences according to region, season, gender, diarrhea, or living conditions. The information on *G. duodenalis* infection in cats is limited, and it has been reported only in certain regions of South Korea: with 3.8% in Jeju (Kwak and Seo, 2020) and 30.7% in Daejeon (Li et al, 2019). The *G. duodenalis* infection rate in this study was higher than 2.3% in the United States (Bouzid et al, 2015), 7% in Denmark (Enemark et al, 2020), 1.4–3.6% in China (Li et al, 2019; Li et al, 2019), and 1.5% in Iran (Karimi et al., 2023). However, it was lower than Brazil at 9.0% (de Oliveira et al, 2021), Australia at 10.1% (Yang et al, 2015), and Turkey at 8.0% (Onder et al, 2021). *Giardia* is the common enteric parasite that causes digestive problems, and infections are frequently confirmed in cats in developed countries (Feng and Xiao, 2011; Pallant et al, 2015). In this study, *G. duodenalis* was the most frequently detected gastrointestinal protozoa parasite, indicating that *G. duodenalis* infections are prevalent in South Korea. Assemblage analysis of *G. duodenalis* using three loci showed infections in assemblages A, B, C, and D. Assemblages AI-2, B18, and D were identified by MLG analysis. *G. duodenalis* has a wide host range compared to many other mammalian species. Each assemblage shows a specific host, and cats have been reported to have zoonotic infections of assemblages A and B, dog-specific assemblages C and D, and cat-specific assemblage F (Appelbee et al, 2005; Feng and Xiao, 2011; Capewell et al, 2021). In a recent study, assemblage F was identified in Jeju, South Korea (Kwak and Seo, 2020). In contrast, assemblages A and B, which are zoonotic subtypes in cats, have been reported in other countries (Li et al., 2019; Li et al., 2019; Enemark et al, 2020; Onder et al, 2021; Procesi et al, 2022). *Giardia* is host-adapted in most species and has a high infection rate (Appelbee et al, 2005; Feng and Xiao, 2011; Wang et al, 2019);. In addition, cats raised by humans have the potential to transmit *Giardia* through contact with people and sharing their environment (Ballweber et al, 2010; Yang et al., 2015; Dixon, 2021). Thus the infections of *G. duodenalis* assemblages A and B in this study are likely to be transmitted to humans. The detection of assemblages C and D (Tangtrongsup et al, 2020) and assemblage D (Palmer et al, 2008; Jaros et al, 2011) in cats has been reported in previous studies. Other animal infections with dog-specific assemblages C and D remain unclear. However, the rare detection in cats, including in this study, indicates the possibility of infection or carriers. Overall, *G. duodenalis* infection in cats is prevalent in South Korea, and it is suggested that cats were infected with the zoonotic type by sharing living environments with humans or other animals.

In this study, *C. felis* and *Cystoisospora felis* were significantly associated with diarrhea. Although it does not cause clinical symptoms in paratenic hosts, it has been reported in previous studies on digestive diseases in cats following infection with *Cryptosporidium* (Lindsay and Zajac, 2004; Rambozzi et al, 2007), *C. felis* (Beser et al, 2015) and *Cystoisospora felis* (Dubey, 2018). However, previous studies have shown no association between the pathological symptoms in the infection of *C. felis* and *Cystoisospora felis* (Ballweber et al, 2010; Lucio-Forster et al, 2010; Dubey et al, 2015; Ito et al, 2017; Li et al, 2019; Meng et al, 2021). Therefore, although an association between *C. felis* and *Cystoisospora felis* infection in cats and diarrhea symptoms was identified in this study, continuous observation with more samples is required. According to the results of the present study, significant differences in infection by living conditions were observed for *C. felis*. Moreover, despite no significant differences, *Cryptosporidium* and *Cystoisospora* infections in companion cats were not identified as subspecies infections other than *C. parvum*. The housing environment of companion animals contributes to the prevalence of parasitic infection (Appelbee et al, 2005). Previous studies have reported a higher rate of *C. felis* infection in stray and shelter cats than in companion cats, although companions also show parasitic infection (Itoh et al, 2013; Li et al, 2019; Li et al, 2021; Taghipour et al, 2021; Karimi et al, 2023). The present results showed that the detection of protozoa parasites varies depending on the habitat of cats and that the low rate of companion cat infection with *Cryptosporidium* and *Cystoisospora* is consistent with the breeding style of companion cats with less outside access in South Korea (Kim et al, 2018). However, the number of fecal samples of companions is very limited compared to the number of samples from stray and shelter cats, and further research through more fecal samples from companion cats is required to identify accurate infection rates. In contrast, the overall detection of *G. duodenalis* in this study was identified in all categories of regions, seasons, gender, fecal state, and living conditions. In previous studies, although the infection rate of *G. duodenalis* was significantly higher in females in Daejeon (Lee et al, 2022), there were no significant differences in infection rates by region, season, gender, diarrhea, age, or living conditions in South Korea (Kwak and Seo, 2020), China (Li et al, 2019; Li et al, 2019), Iran (Karimi et al, 2023), Greece (Kostopoulou et al, 2017), and Italy (Procesi et al, 2022). *G. duodenalis* usually exhibits asymptomatic infections (Lucio-Forster et al, 2010; Certad et al, 2017; Lee et al, 2019). Despite the relatively high detection rate of *G. duodenalis* in cats, this study did not show an association between the infection rate and analytical condition (region, season, gender, fecal states, and living condition), which is consistent with the results of previous studies. The detection rate of *G. duodenalis* was relatively high in companion cats as well as overall infection, and the possibility that it can have clinical symptoms of co-infection with other pathogens as well *as G. duodenalis* itself was suggested in previous studies (Lee et al, 2016; Tangtrongsup et al, 2020). However, it is clear that the number of fecal samples from companion cats secured in this study is small, continuous diagnosis and research of cats will be required.

## Conclusion

The results of the present study showed the detection according to region, gender, diarrhea symptoms, and living conditions of *Cryptosporidium*, *Cystoisospora*, and *G. duodenalis*, which are gastrointestinal protozoa parasites from all provinces of South Korea. Cat-specific *C. felis* and *Cystoisospora felis* were identified most frequently and were associated with living conditions and diarrhea symptoms caused by infection. Moreover, *C. parvum* and *G. duodenalis* assemblages A and B, which are zoonotic subspecies, were detected, suggesting that transmission between humans and cats is possible through environmental sharing. Unexpectedly, other species-specific *C. ryanae*, *Cystoisospora ohioensis*, and *G. duodenalis* assemblages C and D were detected, and further research on cat infections caused by these subspecies is required.

## Data availability statement

Data presented in this study are available upon request from the corresponding author. Representative DNA sequences from the present study were deposited in the GenBank database under the accession numbers OQ598555-OQ598563 for gp60 of C. felis, OQ473126, OQ473172-OQ473185, OQ534549, and OQ534551-OQ534555 for ITS-1 of Cystoisospora, OQ442978-OQ442994 for tpi of G. duodenalis, OQ442958-OQ442969 for b-giardin of G. duodenalis, and OQ442970-OQ442977 for gdh of G. duodenalis.

## Ethics statement

The animal studies were approved by Animal and Plant Quarantine Agency. The studies were conducted in accordance with the local legislation and institutional requirements. Written informed consent was obtained from the owners for the participation of their animals in this study.

## Author contributions

CY: Data curation, Investigation, Methodology, Software, Writing – original draft, Formal Analysis. B-YM: Conceptualization, Writing – review & editing. KL: Conceptualization, Writing – review & editing. SK: Investigation, Writing – review & editing. B-KK: Funding acquisition, Writing – review & editing. M-HH: Formal Analysis, Writing – review & editing, Data curation.
